# Tobacco prevention policies in west-African countries and their effects on smoking prevalence

**DOI:** 10.1186/s12889-015-2562-z

**Published:** 2015-12-08

**Authors:** Volker Winkler, Yong Lan, Heiko Becher

**Affiliations:** Institut für Public Health, Universitätsklinikum Heidelberg, Heidelberg, Germany; Institut für Medizinische Biometrie und Epidemiologie, Universitätsklinikum Hamburg-Eppendorf, Hamburg, Germany

**Keywords:** Smoking prevalence, Policy measures, Tobacco control, MPOWER, West-Africa

## Abstract

**Background:**

The WHO Framework Convention on Tobacco Control was shown to effectively lower smoking prevalence in in high income countries, however knowledge for low and middle income settings is sparse. The objective of this study was to describe WHO MPOWER policy measures in thirteen West-African countries and to investigate their correlation with smoking prevalence.

**Methods:**

Age-standardized smoking prevalence data and policy measures were collected from various WHO reports. For analysis MPOWER measures from 2008 and 2010, were combined with prevalence data from 2009 and 2011. Multiple linear regression models were set up.

**Results:**

In West-Africa mean smoking prevalence was approximately 20 % among males and approximately 3 % among females. Policy measures were mostly at a middle or low level. Regression analysis showed that tobacco cessation programs, health warnings on cigarettes, and higher price of cigarettes were negatively correlated with smoking prevalence. Significant effects were observed for only one policy measure (tobacco cessation programs) and only within the male population where smoking prevalence is generally higher.

**Conclusions:**

Tobacco control policies are enforced at relatively low levels in West-African countries. However, improving tobacco control policy implementation according to the WHO Framework Convention on Tobacco Control should assist in the reduction of smoking prevalence in African countries, thereby counteracting pro-smoking initiatives set forth by the tobacco industry.

**Electronic supplementary material:**

The online version of this article (doi:10.1186/s12889-015-2562-z) contains supplementary material, which is available to authorized users.

## Background

Diseases related to tobacco consumption rank among the most preventable causes of deaths and disability among adults in the world today [1]. The 2011 World Health Organization (WHO) report on the Global Tobacco Epidemic highlighted that tobacco use kills nearly 6 million people and causes several hundred billion dollars of economic damage worldwide each year [2]. By the year 2025, the total number of tobacco users is expected to increase further and three times as many people are estimated to die from smoking-related diseases. More than 80 % of these premature deaths are predicted to occur in low and middle income countries, where health facilities and services are inadequately equipped to cope with the demands presented by the tobacco epidemic [3].

Most African nations still remain in the early stages of the tobacco epidemic, with an overall lower smoking prevalences and lower smoking intensities than observed in other parts of the world [4, 5]. However, it is important to note that smoking prevalence values vary significantly between individual countries.

For example, Guinea with a smoking prevalence of 52 % in 2002 ranked among the top 10 countries with the highest smoking prevalence worldwide, whereas Ghana and Senegal both had significantly lower reported smoking prevalences of <5 % [2, 6].

Longitudinal data for Africa are sparse and few reliable data on smoking prevalence are available in the literature. Estimates on longitudinal trends of tobacco smoking in Africa show a relatively low and stable age-standardized prevalence of about 16 % among males and 3 % among females [6]. However, indicators of increasing activities from the tobacco industry in African countries have been observed, which may lead to a higher smoking prevalence in the absence of proper tobacco control policy implementation [7].

In response to the globalization of tobacco epidemic, the WHO Framework Convention on Tobacco Control (FCTC), was adopted by the World Health Assembly in May 2003 and entered into force in February 2005. In 2008, the WHO introduced the MPOWER package in order to assist countries in the fulfillment of WHO FCTC obligations. This policy package is intended to scale up global tobacco control, and covers six measures to assist in the country-level implementation of effective interventions to reduce the demand for tobacco: monitoring tobacco use and prevention policies, implement smoke free laws, offer help to quit tobacco use, warn about tobacco dangers, advertisement bans, and raise taxes on tobacco [8]. MPOWER measures reported by the WHO allow for both standardized comparisons between countries and within countries themselves over a predefined period of time.

While it is known from studies in industrialized countries that public health efforts can be successful in reducing smoking prevalence, the effect of such action has not yet been investigated in most West-African countries [9]. In this ecological study, we will use WHO’s MPOWER measures to describe tobacco control policies in thirteen West-African countries, and analyze their relation to country-wide smoking prevalence data to strengthen successful tobacco control policy in West-Africa.

## Methods

### Data sources

We used sex-specific age-standardized smoking prevalence estimates of thirteen West-African countries (Benin, Burkina Faso, Cape Verde, Côte d’Ivoire, Gambia, Ghana, Guinea, Mali, Mauritania, Niger, Nigeria, Senegal and Sierra Leone) from WHO reports years 2009 and 2011 [2, 10]. The remaining three West-African countries (Guinea-Bissau, Liberia and Togo) were excluded from the study due to missing data. These WHO estimates are based on different national or subnational surveys, which were constructed for the purpose of comparing adult tobacco use prevalence across multiple time periods within the same country [10]. Age-standardized smoking prevalence for Burkina Faso in 2011 was derived from a global estimation study on tobacco smoking [6]. Smoking prevalence was defined as smoking at the time of the survey including daily and non-daily use of any form of tobacco, such as cigarettes, cigars, pipes, etc. excluding smokeless tobacco. A detailed description of all underlying surveys is given in the Appendix 1.

Information pertaining to smoking prevention policies are based on reports to the Conference of the Parties (COP) published by the WHO for the years 2008 and 2010 [2, 8]. The MPOWER measures smoke-free places, tobacco treatment, health warning, advertisement ban, and national agency were then categorized into four groups according to the WHO standard as shown in Table 1. Additionally, cigarette price in international dollars adjusted for purchasing power was extracted from the reports for all countries. All data used for the study are freely available from the cited WHO documents.

**Table 1:** Coding of MPOWER measures and their observed distribution in West-African countries

Variable	Level	Description	Code^a^	Observed distribution	
				2008	2010
Current smoking prevalence		Prevalence of tobacco smoking	[%]		
			Mean	11.7 %	13.1 %
			Median	8.6 %	9.5 %
			Range	(<0.1–38.6 %)	(<0.1–48.0 %)
Number of smoke-free places (smoke-free places)	I	≤2 public places completely smoke-free	0	6 (47 %)	8 (61 %)
	II	3–5 public places completely smoke-free	1	3 (23 %)	3 (23 %)
	III	6–7 public places completely smoke-free	2	2 (15 %)	1 (8 %)
	IV	All public places completely smoke-free	3	2 (15 %)	1 (8 %)
Level of cessation service (tobacco treatment)	I	None	0	3 (23 %)	3 (23 %)
	II	Some cessation service not cost-covered	1	6 (47 %)	7 (54 %)
	III	At least one service is cost-covered	2	4 (30 %)	3 (23 %)
	IV	National quit line, services cost-covered	3	0 (0 %)	0 (0 %)
Level of health warning labels (health warning)	I	No warnings	0	9 (70 %)	10 (77 %)
	II	Medium size warnings with missing characteristics	1	4 (30 %)	3 (23 %)
	III	Medium size warnings	2	0 (0 %)	0 (0 %)
	IV	Large warnings	3	0 (0 %)	0 (0 %)
Level of bans on advertising, promotion and sponsorship (advertisement ban)	I	No ban	0	4 (30 %)	5 (38 %)
	II	Ban on TV, radio and print media	1	5 (39 %)	2 (15 %)
	III	Ban on TV, radio and print media, and on some direct and/or indirect advertising	2	3 (23 %)	5 (39 %)
	IV	Ban on all forms of advertising	3	1 (8 %)	1 (8 %)
Level of tobacco control program (national agency)	I	No national agency on tobacco control	0	2 (15 %)	2 (15 %)
	II	Existence of national agency	1	5 (39 %)	3 (23 %)
	III	National agency with ≤5 staff members	2	2 (15 %)	4 (31 %)
	IV	National agency with >5 staff members	3	4 (31 %)	4 (31 %)
Price of cigarettes (adjusted price)		Price of 20-cigarette pack in dollars (at purchasing power parity)	[$]		
			Mean	$2.08	$2.04
			Median	$2.3	$2.3
			Range	($1.00–2.95)	($0.86–2.94)

^a^As used in regression

### Statistical methods

Multiple linear regression models were used to examine the correlation of the different MPOWER measures on smoking prevalence. Smoking prevalence percentage was used as a continuous dependent variable and coded MPOWER measures were set as independent variables. Since percentages fall in a sufficiently narrow range it is appropriate to model these directly with linear regression if the [11]. This is the case with our data (see Discussion). The MPOWER measures from 2008 were combined with prevalence estimates for 2009, and similarly the measures from 2010 with prevalence estimates from 2011. Therefore, all countries provided two observations. Since the observations were not independent, the inference was done by bootstrapping the p-values, where the observations from one country were taken as the bootstrap unit [12]. The bootstrap sample size was taken as 1000. The full dataset is given in the appendix. Due to very low smoking prevalence among women, the analysis was done for both sexes separately. For sensitivity analyses, we also fit the models for the two years separately. Note that with the available data we are not able to make any statement on a causal relation between smoking prevalence and these potentially influential factors.

## Results

Extracted policy measures and smoking prevalence data are presented in Table 2. The average smoking prevalence among West-African males (unweighted mean) was 20.9 %, while it was only 3.9 % among females. There was no clear trend towards an increasing prevalence between 2009 and 2011. When looking at individual countries the decrease in male smoking prevalence was seen for Guinea (-2.1 %) and Côte d’Ivoire (-1.3 %) whereas the strongest increase was seen for Sierra Leone (+9.4 %) followed by Benin (+5.8 %) and Ghana (+3.4 %). Among females smoking prevalence decreased strongest for Burkina Faso (-4.0 %), but increased for Sierra Leone (+11.8 %) followed by Côte d’Ivoire (+5.4 %) and Ghana (+4.4 %).

**Table 2:** Data on policy measures and smoking prevalence in West-African countries

Country	Smoking prevalence				Policy intervention					
	Year prevalence	Males	Females	Year policy	Smoke-free places	Tobacco treatment	Health warning	Advertisement	National agency	Adjusted price [$]
Benin	2009	15.2 %	1.5 %	2008	1	1	1	2	2	2.18
Benin	2011	21.0 %	3.0 %	2010	1	1	1	2	2	2.13
Burkina Faso	2009	18.2 %	8.0 %	2008	1	2	0	0	1	2.44
Burkina Faso	2011	20.0 %	4.0 %	2010	3	1	0	2	1	2.33
Cape Verde	2009	13.5 %	3.4 %	2008	0	2	0	1	1	2.95
Cape Verde	2011	14.0 %	3.0 %	2010	0	2	0	1	2	2.78
Côte d’Ivoire	2009	17.3 %	3.6 %	2008	0	1	1	1	3	2.28
Côte d’Ivoire	2011	16.0 %	9.0 %	2010	0	1	0	0	3	2.29
Gambia	2009	31.3 %	2.7 %	2008	2	0	0	2	1	1.31
Gambia	2011	32.0 %	3.0 %	2010	0	0	1	2	1	1.19
Ghana	2009	10.6 %	2.6 %	2008	0	1	1	1	2	2.94
Ghana	2011	14.0 %	7.0 %	2010	0	1	0	1	2	2.94
Guinea	2009	25.1 %	1.9 %	2008	3	1	0	2	3	1.00
Guinea	2011	23.0 %	2.0 %	2010	2	1	0	2	3	0.86
Mali	2009	28.5 %	2.2 %	2008	2	1	0	1	1	2.70
Mali	2011	28.0 %	2.0 %	2010	0	1	0	2	2	2.56
Mauritania	2009	29.4 %	4.1 %	2008	0	0	0	0	0	2.59
Mauritania	2011	29.0 %	4.0 %	2010	0	0	0	0	0	2.78
Niger	2009	8.9 %	0.0 %	2008	3	1	1	3	1	1.69
Niger	2011	9.0 %	0.4 %	2010	1	1	1	3	1	1.57
Nigeria	2009	10.5 %	2.6 %	2008	1	2	0	0	3	2.35
Nigeria	2011	10.0 %	2.0 %	2010	1	2	0	0	3	2.66
Senegal	2009	15.6 %	0.0 %	2008	0	2	0	1	3	1.48
Senegal	2011	16.0 %	0.4 %	2010	0	2	0	0	3	1.50
Sierra Leone	2009	38.6 %	8.2 %	2008	0	0	0	0	0	1.17
Sierra Leone	2011	48.0 %	20.0 %	2010	0	0	0	0	0	0.99

The distribution of observed policy intervention is shown in Table 1. Overall average levels of all interventions were at a middle or low level (level 1 or 2). 60 % of the price for a pack of 20 cigarettes was above 2.00 international dollars. Compared with policy interventions in 2008, some smoking prevention policies have been weakened in 2010, e.g. smoke-free places and cessation services. Health warning remained at a low level. Moreover, the cigarette price adjusted for purchasing power decreased from 2.08 international dollars in 2008 to 2.04 international dollars in 2010.

**Fig. 1:**
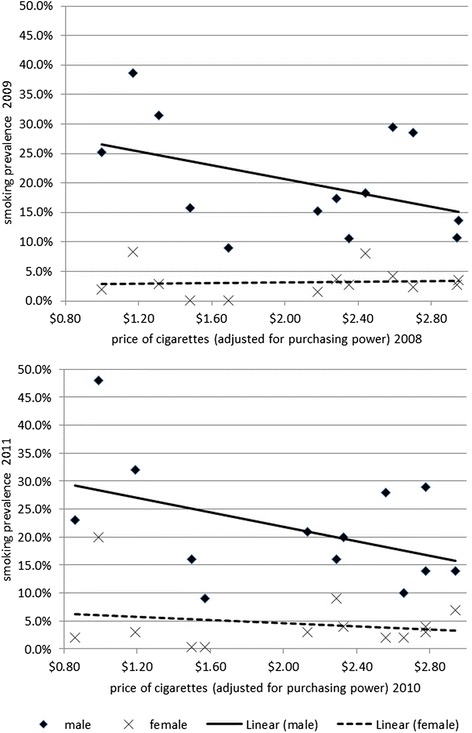
Correlation of cigarette price (adjusted for purchasing power) and smoking prevalence for the time periods 2008/2009 (top) and 2010/2011 (bottom)

Figure 1 demonstrates the negative correlation between cigarette price and the smoking prevalence among males. No correlation was observed for females. Similar conclusion can be drawn through the analysis of other policy interventions and smoking prevalence, e.g. smoke-free places, health warning, etc. (results shown in Additional file 1).

The multiple linear regression analysis shown in Table 3 show that all policy indicators are independently correlated with smoking prevalence. All indicators resulted in negative coefficients. Since the dataset was small, the bootstrapped p-values were mostly not significant at the commonly used α = 0.05 level and the estimates must be interpreted accordingly. The model variables explained a large part of the variation of smoking prevalence among males (R^2^ = 0.8). The model for females revealed similar results, however, due to the low prevalence of smoking in females, the effects were lower and the estimates had higher p-values.

**Table 3:** Coefficients of multiple linear regression model

	Males		Females	
Independent variables	β	p-value*	β	p-value*
(Constant)	0.458	0.00	0.119	0.00
Smoke-free places	−0.011	0.42	−0.003	0.57
Tobacco treatment	−0.078	0.02	−0.011	0.56
Health warning on tobacco products	−0.074	0.12	−0.042	0.42
Advertisement restricted/not allowed	−0.009	0.60	−0.020	0.15
Tobacco Control program	−0.018	0.30	−0.001	0.51
Price [$] (adj. purchasing power)	−0.048	0.14	−0.013	0.72
Model R^2^	0.80		0.47	

*Empirical bootstrap p-values

In order to analyze the impact of using several observations per country we performed a sensitivity analysis by limiting the dataset to one observation per country. Results show that although the estimated coefficients for the policy indicators change little, p-values increase strongly due to the smaller number of observations.

## Discussion

The study results gave an overview of recent smoking prevalence data and tobacco control policies implemented in West-African countries. Smoking prevalence was not high, but the analysis of tobacco control policies revealed that most countries have not yet achieved full FCTC implementation. Furthermore, it was shown that policy measurements suggested by the WHO correlated with reduced smoking prevalence in Western Africa. Highest impacts on prevalence were seen for tobacco cessation programs, health warning and cigarette price.

A recent study on the relation of smoking prevalence and policy interventions measured in a combined policy score also showed the reducing effect of policy on smoking prevalence in 59 countries worldwide [9]. However, this study only included three West-African countries.

The availability and implementation of tobacco cessation programs as recommended in Article 14 of the FCTC appear to be lowly prioritized, particularly in lower-income countries [13]. Although nicotine replacement therapies were proven to be effective at increasing the success rate of smoking cessation and therefore at lowering smoking prevalence, potential harm and benefits of alternative nicotine delivery systems are still under investigation [14, 15].

Comprehensive tobacco advertising bans as recommended in Article 13 of the FCTC were proven to be effective at both preventing tobacco initiation as well as encouraging smokers to quit. Advertising bans seem even more effective in low and middle income countries than in high income countries [16].

Sufficient evidence has shown that the cigarette price strongly correlates with smoking prevalence, and that raising cigarette retail price (as recommended in Article 6 of the FCTC) reduces cigarette consumption significantly [2]. Other studies have suggested that the relative contribution of cigarette price on the reduction of male smoking prevalence is above 60 % [17, 18]. This heavy influence of cigarette price on the reduction of smoking prevalence may function in two different ways: current and future smokers may be prevented from smoking altogether, and/or preexisting smokers may reduce their overall cigarette intake [19, 20]. Although cigarette pricing is an important strategy of tobacco control, the absolute value of cigarette price may be misleading without considering affordability [21]. Compared with western-countries, the affordability of cigarettes in many West African nations has increased in the past decades due to higher income obtained [21, 22].

As mentioned before, smoking patterns between men and women in West Africa differ. Therefore, effective smoking prevention policies require consideration of gender differences. According to the estimates from the regression model without taking into account p-values, health warnings seem to have the largest impact on female smokers, followed by advertisement bans. In contrast, tobacco cessation programs seem to be the policy factor with the largest impact on male smoking prevalence, followed by health warning labels and cigarette price. Interestingly the WHO has reported that women are increasingly targeted by the tobacco industry in many countries [10]. Therefore, it is important to ensure that particular political smoking reduction strategies take into account gender differences.

The data for our study are sparse. More information on time trends both with respect to smioking prevalence as well as prevention measures would have strengthened our analysis. Therefore, we unfortunately could not explore a dynamic relationship between policy implementation and smoking prevalence in this study. Some countries have not established a functional surveillance system to monitor tobacco use and tobacco control measures.

As this study is an ecological study it exclusively relies on aggregated data at the country level and therefore suffers well known limitations related to such analyses such as the ecological fallacy. Other factors may exist which could partly explain differences in country-specific smoking prevalences. The study is not large, and therefore the confidence intervals are wide. The observed smoking prevalences, with very few exceptions, fall within a relatively narrow range from 10 to 30 % in males and 0 to 20 % in females. We therefore considered the linear regression approach an acceptable modeling system to use. The use of more multiple observations per country is taken into account by using a bootstrap approach to estimate the confidence intervals. Results of the sensitivity analysis showed there is only very limited impact on the regression estimates by using several observations per country. Due to the limited number of observations we treated the categorical policy levels of all policy interventions as continuous variables within the multivariate regression model. This means that the given estimates referred to the average change per policy level or per one dollar in price. However, in reality, the impact on smoking prevalence may be different between for example policy level 1 to 2 and that of policy level 2 to 3. To take into account such possible nonlinear relationships we performed the multivariate linear regression by using fractional polynomials, but this resulted in a standard linear regression model. Overall, we are confident our analyses are reasonable.

Policy measurements may also be biased by different qualities of implementation. Furthermore, we cannot rule out that extracted data is mixed with other political factors, which are not directly measured but may influence the effects on the smoking prevalence.

## Conclusion

Many West-African governments have implemented some degree of tobacco control policy in response to health and economic threats presented by increased tobacco consumption. However, such tobacco control policies require urgent strengthening in order to combat recently enhanced activities of the tobacco industry in West-African countries [23, 24]. Results of this study support the hypothesis of smoking prevalence reduction by the implementation of regulated tobacco control policy, and hence may provide evidence necessary to support future governmental decisions regarding the strengthening of tobacco control policy.

## Appendix 1

**Table 4:** Sources of WHO smoking prevalence data in West-African countries

Country	Survey year	Age group (years)	Sample size (n)	Type	Representativeness
Benin	2008	25–64	2708	WHO STEPS	National
Burkina Faso	2003	18+	4948	World health survey	National
Cape Verde	2007	25–64	1762	WHO STEPS	National
Côte d’Ivoire	2005	15–64	4756	WHO STEPS	Subnational
	2012	Male: 15–59 Female: 15–49	19559	Demographic and health survey	National
Gambia	2010	25–64	4111	WHO STEPS	National
Ghana	2008	Male: 15–59 Female: 15–49	9484	Demographic and health survey	National
Guinea	2009	15–64	2491	WHO STEPS	Subnational
Mali	2007	15–64	2810	WHO STEPS	Subnational
Mauritania	2006	15–64	1971	WHO STEPS	National
Niger	2007	15–64	2191	WHO STEPS	National
Nigeria	2008	15–59	48871	Demographic and health survey	National
Senegal	2003	18+	3465	World health survey	National
Sierra Leone	2009	25–64	5483	WHO STEPS	National

## Additional file

Additional file 1:
**Correlation of policy interventions and smoking prevalence for the time periods 2008/2009 (left) and 2010/2011 (right).** (JPEG 3067 kb)
