# Supplementary figures and images for: Tobacco prevention policies in west-African countries and their effects on smoking prevalence
Source: BMC Public Health. 2015 Dec 8;15:1216. doi: 10.1186/s12889-015-2562-z (PMC4673866; doi:10.1186/s12889-015-2562-z)

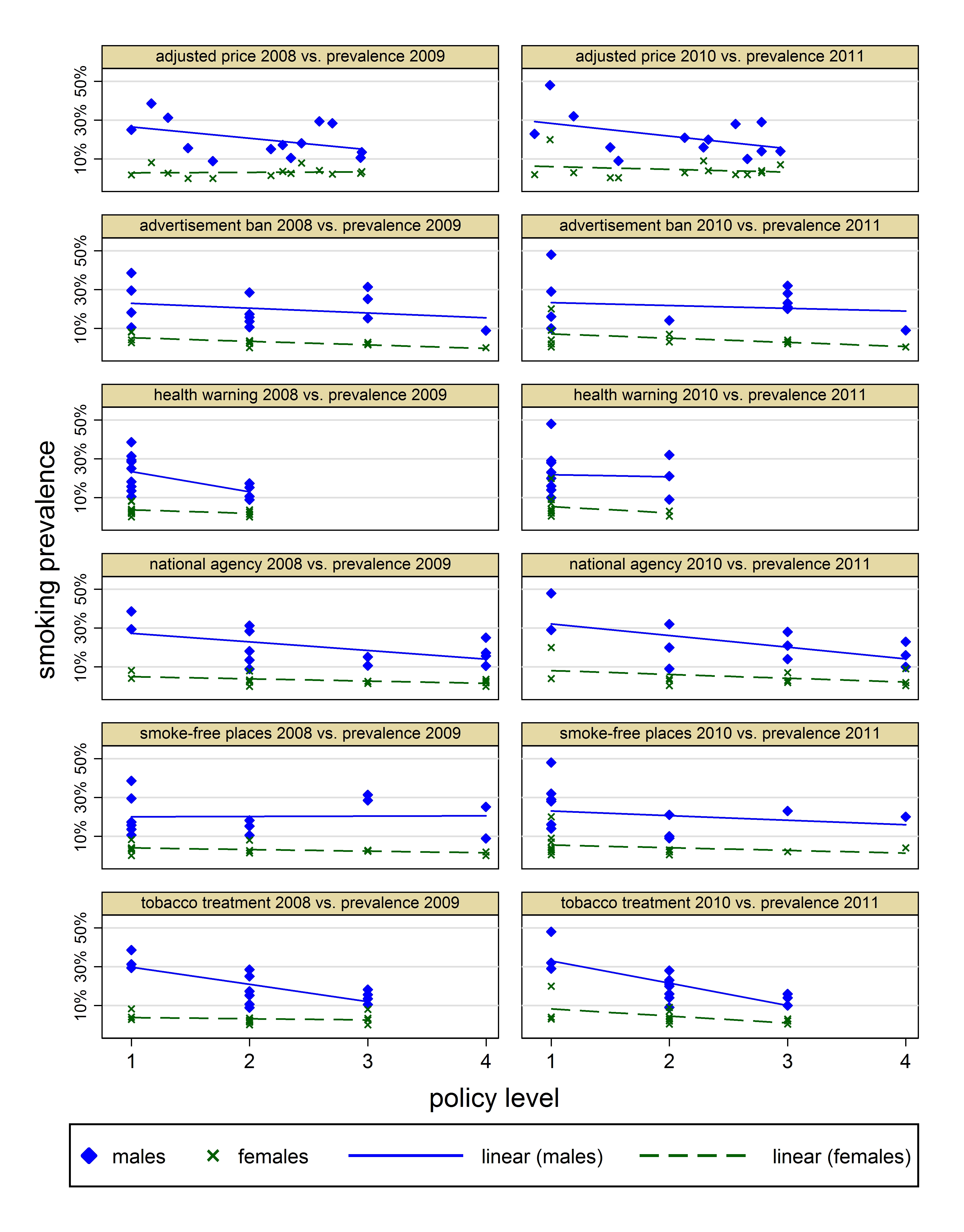

Supplement: Additional file 1: — Correlation of policy interventions and smoking prevalence for the time periods 2008/2009 (left) and 2010/2011 (right). (JPEG 3067 kb) [file 12889_2015_2562_MOESM1_ESM.jpeg]
